# Detection of arboviruses in *Aedes aegypti* through transovarian analysis: A study in Goiânia, Goiás

**DOI:** 10.1590/0037-8682-0280-2023

**Published:** 2024-02-23

**Authors:** Diego Michel Fernandes Da Silva, Juliana Santana de Curcio, Lívia do Carmo Silva, Flávia Barreto de Sousa, Carlos Eduardo Anunciação, Silvia Maria Salem-Izacc Furlaneto, Victoria Porto Sandre Missiatto Silva, Marco Túlio Antônio Garcia-Zapata, Elisângela de Paula Silveira-Lacerda

**Affiliations:** 1 Universidade Federal de Goiás, Centro de Estudos e Pesquisas sobre Agentes (Re)emergentes e Unidade Sentinela e Centro de Referência em Medicina Internacional e de Viagens, Goiânia, GO, Brasil.; 2 Instituto de Patologia Tropical e Saúde Pública, Universidade Federal de Goiás Brasil, Goiânia, GO, Brasil.

**Keywords:** Arboviruses, RT-qPCR, Vertical transmission, Monitoring

## Abstract

**Background:**

Arboviral diseases are a group of infectious diseases caused by viruses transmitted by arthropods, mainly mosquitoes. These diseases, such as those caused by the dengue (DENV), Zika (ZIKV), chikungunya (CHIKV), and yellow fever (YFV) viruses, have a significant impact worldwide. In this context, entomological surveillance plays a crucial role in the control and prevention of arboviruses by providing essential information on the presence, distribution, and activity of vector mosquitoes. Based on entomological surveillance, transovarian transmission provides information regarding the maintenance and dissemination of arboviruses. The objective of this study was to detect these arboviruses in Goiânia, Goiás, and analyze the occurrence of transovarian transmission.

**Methods:**

*Aedes aegypti* eggs were collected from different regions of Goiânia and cultivated under controlled laboratory conditions until the emergence of adult mosquitoes. Adult females were grouped into pools containing their heads and thoraxes. These pools were subsequently evaluated using reverse-transcription quantitative polymerase chain reaction (RT-qPCR) assay.

**Results:**

A total of 157 pools (N=1570) were analyzed, with two pools testing positive for CHIKV and one pool testing positive for ZIKV, indicating that the offspring resulting from transovarian transmission are potentially infectious.

**Conclusions:**

In summary, the demonstration of the vertical transmission mechanisms of CHIKV and ZIKV in *A. aegypti* serves as an alert to health authorities, as these diseases are still underreported, and their primary urban vector has likely acquired this capacity, contributing to the dissemination of these infections.

## Introduction

Arboviral diseases are a group of infectious diseases caused by viruses transmitted by arthropods that are primarily members of the Culicidae family. These diseases, including those caused by the dengue (DENV), Zika (ZIKV), yellow fever (YFV) (Flaviviridae family), chikungunya (CHIKV) (Togaviridae family), and oropouche (OROV) (Peribunyaviridae family) viruses, pose a significant challenge to public health worldwide^1^. In addition to impacting human health, arboviral diseases have a significant impact on society and the economy^2^. The social impact of arboviral diseases is marked by human suffering caused by debilitating symptoms, such as high fever, intense joint pain, and skin rashes^1^ ^,^ ^3^, as well as severe complications, such as Guillain-Barré syndrome^4^ and ZIKV-induced microcephaly in neonates^5^. Some arboviral diseases can be fatal, such as dengue hemorrhagic fever (DHF) and dengue shock syndrome (DSS)^6^ ^,^ ^7^. Vulnerable populations, including children, the elderly, and individuals with preexisting health conditions, are the most affected. In addition, arboviral diseases impose a significant burden on healthcare systems, individuals, and their families. Direct costs include medical treatment, hospitalization, and long-term care for patients with sequelae. There are also indirect costs, such as loss of productivity at work due to illness or disability, which negatively impact the local and national economies^8^.

Mosquitoes of the genus *Aedes* are the primary vectors of arboviral diseases. *Aedes aegypti* and *Aedes albopictus* are the main vectors of DENV, ZIKV, and CHIKV^9^ ^,^ ^10^. They are highly adapted to urban environments and are primarily bred in artificial containers that collect stagnant water, such as tires, plant pots, and household containers^11^. Additionally, they are capable of transovarial transmission^12^ ^,^ ^13^. The larvae that emerge from the eggs are already infected and become adult mosquitoes capable of transmitting the virus to other hosts, contributing to the persistence of arboviruses in urban areas, even during periods of low incidence of human cases. This makes controlling these diseases more challenging, because continuous transmission by infected mosquitoes from the larval stage can perpetuate viral circulation^14^ ^,^ ^15^.

Entomological surveillance focuses on these mosquitoes and plays a crucial role in the control and prevention of arboviral diseases by providing essential information on the presence, distribution, and activity of vector mosquitoes^16^. Through the placement of traps to capture adult mosquitoes and the inspection of breeding sites to analyze eggs and larvae for the presence of arboviruses, it is possible to identify areas with higher infestation, determine the population density of vectors, and monitor the presence of arboviruses in mosquitoes^17^. Continuous and comprehensive entomological surveillance is essential to monitor vector activity, evaluate the effectiveness of control measures, and provide crucial information for planning and implementing strategies to prevent and control arboviral diseases^18^.

Among the surveillance approaches used, surveillance of the transovarial transmission in vector mosquitoes is noteworthy. This type of surveillance enables the identification and monitoring of arboviruses in mosquito populations from the larval stage to the adult phase when they can transmit the disease. A systematic collection of eggs, larvae, and mosquitoes allows the identification of sites where transovarial transmission occurs and the estimation of the viral infection rate in these populations^19^. Surveillance of transovarial transmission in mosquitoes is a strategic and complementary approach to conventional entomological surveillance, contributing to the interruption of the viral transmission chain and the effective control of arboviral diseases^20^. 

Considering that there was a 211% increase in confirmed DENV cases, a 430% increase in CHIKV cases, and a 364.71% increase in ZIKV cases from 2021 to 2022, as reported in the epidemiological bulletin of the state of Goiás, it is possible that this increase is related to transovarial transmission^21^. Therefore, the objective of this study was to monitor the arboviruses DENV, CHIKV, ZIKV, and OROV through the analysis of transovarial transmission occurrence in *A. aegypti* mosquitoes in the city of Goiânia, Goiás.

## Methods

● Study area and collection of eggs

**FIGURE 1:**
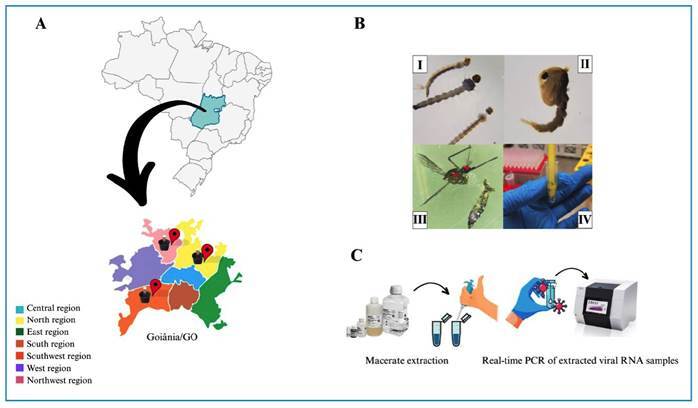
Collection regions and steps carried out in this study. In general, this work included the following steps: **(A)** capturing *A. aegypti* eggs in the North, Northwest and Southwest regions of Goiânia. **(B)** (I) Development of larvae; (II) development of pupae; (III) identification of mosquitoes, gender differentiation, and separation of the head and thorax from the abdomen; (IV) and maceration of the pools. **(C)** Extraction of the macerated pools and RT-qPCR of extracted samples.

*A. aegypti* eggs were laid in the city of Goiânia, Goiás (Latitude: −16.6799 and Longitude: −49.255). It is the most populous city in the central region of Brazil, with more than 1.5 million inhabitants. The semi-humid tropical climate contributes to the proliferation of the vector of arboviruses, *A. aegypti*, causing Goiânia to register an increase in cases of arboviruses annually, with regions of the municipality in a state of alert for epidemics. Previous studies on transovarian transmission in *A. aegypti* were carried out in the city where the Mayaro virus (MAYV) was detected in vectors close to Basic Health Units in the municipality^22^. In view of this, three regions of the city-north (N), southwest (SW), and northwest (NW)-were chosen for the collection of *A. aegypti* eggs, for the analysis of transovarial transmission in this vector (Figure 1 **).** The collection was conducted by agents of the Sanitary Surveillance of the State of Goiás (SVS/GO) from January to September 2022, ovitraps were used to capture the eggs. 

● Development of mosquitoes, assembly of pools, and extraction of viral RNA

The collected eggs were added to containers containing distilled water and nutrients until the larvae hatched. The larvae were collected using a sieve and placed in 2 L PET bottles containing sterile distilled water and cat food, which were later sealed with cotton. Adult mosquitoes were captured by suction using an aspirator and placed directly in falcon tubes lined with filter paper and immediately stored in a −80 °C freezer until the pools were assembled. All reagents used during mosquito manipulation were tested using reverse-transcription quantitative real-time PCR (RT-qPCR) to determine the presence of internal contamination.

Morphological identification was performed at the genus and species levels as described by Forattini et al.^23^. Adult mosquitoes were sexed by observing characteristics such as the plumage of the insects’ antennae, as shown in Supplementary Figure 1. The females were separated and organized into pools of 10 individuals containing the head and thorax for analysis of the salivary gland and stored in 1.5 mL tubes (Axygen, USA). Mosquito pools were macerated with a plastic pestle in 300 µL of H2O MilliQ autoclaved directly in tubes, and the macerate was centrifuged for 2 min at 500×*g*. The supernatant was collected and transferred to new tubes and stored at −80 °C until viral RNA extraction. Viral extraction was performed with a MagMAX™ Viral/Pathogen Nucleic Acid Isolation Kit (Thermo Fisher, Scientific, Waltham, Massachusetts, USA), according to the manufacturer’s instructions.

● Molecular detection of DENV, CHIKV, ZIKV, and OROV

The detection of arboviruses DENV, ZIKV, and CHIKV was performed using the primers and probes synthesized according to the CDC (Center for Disease Control and Prevention, USA) and employed the GoTaq® Probe 1-Step RT-qPCR system (Promega, WI, USA). The primers for OROV were based on the methods described by Rojas et al.^24^ (Table 1). A set of primers and probes that amplified the actin gene of *Aedes* spp. were used as endogenous controls for RT-qPCR.

**TABLE 1:** Sequence of primers/probes for identification of DENV, CHIKV, ZIKV, and OROV arboviruses and the endogenous control actin gene of *Aedes* spp.

Target	Primer forward	Primer reverse	Probe
Actin	5'GACYGACTACCTGATGAAGATC 3'	5'GTTCATAAGACTTCTCCAGGG 3'	5' SUNCTGGACTTCGAGCAGGAAATGG 3' IowaBlack
OROV	5' GACAAGTSCTCAATGCTGGTGT 3'	5'CGTTGTCCGGSACTGGATT 3'	5'TGGTTGACCTYACTTTTGGTGGGGT 3' FAM/ZEN ^TM^ IBFQ
DENV	5'-GGTTAGAGGAGACCCCTCCC-3'	5'GAGACAGCAGGATCTCTGGTCT-3'	5'/56FAM/AAACAGCAT/ZEN/ATTGACGCTGGGA/3iABkFQ/3'
ZIKV	5'-CCGCTGCCCAACACAAG-3'	5'CCACTAACGTTCTTTTGCAGACAT-3'	5'-/56FAM/AGCCTACCT/ZEN/TGACAAGCAGTCAGACACTCAA/3lABkFQ/-3'
CHIKV	5'-AAAGGGCAAACTCAGCTTCAC-3'	5'GCCTGGGCTCATCGTTATTC-3'	5'-/56FAM/CGCTGTGAT/ZEN/ACAGTGGTTTCGTGTG/3lABkFQ/-3'

**OROV:** oropouche virus; **DENV:** dengue virus; **ZIKV:** Zika virus; **CHIKV:** chikungunya virus.

Gblocks (Integrated DNA Technologies, Coralville, IA, USA), synthetic DNA fragments from DENV (GenBank accession number OK040058.1), CHIKV (GenBank accession number MW581885.1), ZIKV (GenBank accession number OK054487.1), and OROV (GenBank accession number HQ830492.1), were designed and used as positive controls to establish standard curves for absolute quantification by RT-qPCR. Subsequently, a second Gblock was designed to contain primer-binding regions for OROV and the actin gene, as shown in Supplementary Table 1.

The total volume of RT-qPCR was 10 μL (5 μL GoTaq® Probe 1-Step RT-qPCR System kit, 0.2 μL Go Script RT Mix for 1-Step RT-qPCR, 0.8 μL Nuclease-Free Water, 0.5 primers/probe, and 2.5 µL of RNA) (Promega, Madison, Wisconsin, USA). Nuclease-free water for molecular biology (Sigma-Aldrich, EUA) was used as a negative control. RT-qPCR was performed using an AriaMX Real-Time PCR System (Agilent, CA, USA). The amplification program consisted of one cycle at 45 °C for 15 minutes for reverse transcription and one cycle at 95 °C for 2 minutes for reverse transcriptase denaturation and DNA polymerase activation, followed by 40 cycles at 95 °C for 15 seconds and 60 °C for 1 min for denaturation and amplification. Fragments of both Gblocks were used as positive controls and to construct standard curves for absolute quantification of the RT-qPCR reactions. The standard curve for each specific arbovirus or actin gene was constructed using a serial log^10^ dilution ranging from 10^5^ to 1 copy/reaction, and each reaction was performed in duplicate. A standard curve was used to determine the number of copies of viral genetic material present in the pools of mosquitoes or the endogenous control gene, actin. Pools with Cq values ​​equal to or below 34.30 for actin^25^, 31.17 for DENV, 33.49 for ZIKV, 31.66 for CHIKV^22^, and 34.54 for OROV were considered positive (data not shown). Pools with Cq values above the limit of detection were considered negative. 

## Results and Discussion

A total of 1570 *A. aegypti* females were captured in the three major regions of Goiânia and distributed in 157 pools. This resulted in 83 (52.9%) pools from the northwest region, 48 (30.6%) from the southwest region, and 26 (16.6%) from the north region (data not shown). Two samples from the north region tested positive for CHIKV. The southwest region had a positive pool for ZIKV. The northwest region did not show any positive pools. DENV and OROV were not detected in this study. A total of 26 ovitraps were used in this study, with 14 in the northwest region, 7 in the southwest region, and 4 in the northern region (Table 2).

**TABLE 2:** Location coordinates of ovitraps distributed in the southwest, northwest, and north regions of Goiânia.

Region	Nº ovitraps	X^a^	Y^b^	CHIKV	ZIKV	Cq	Number of copies^c^
**Southwest**	8	671823,45	8144543,67	-	-	No cq	ND
	9	672014,84	8144326,97	-	-	No cq	ND
	10	671744,45	8144305,96	-	+	28.48	783
	11	671894,29	8143982,35	-	-	No cq	ND
	12	672339,7	8144028,63	-	-	No cq	ND
	13	672542,9	8143732,42	-	-	No cq	ND
	14	672023,68	8143942,31	-	-	No cq	ND
**Northwest**	26	679788,69	8162192,31	-	-	No cq	ND
	27	679825,04	8161881,73	-	-	No cq	ND
	28	679486,96	8162145,81	-	-	No cq	ND
	29	679466,46	8161650,16	-	-	No cq	ND
	30	679185,51	8161458,6	-	-	No cq	ND
	31	679564,92	8161217,67	-	-	No cq	ND
	32	679274,36	8160818,31	-	-	No cq	ND
	33	678890,45	8160983,68	-	-	No cq	ND
	34	679417,77	8160375,21	-	-	No cq	ND
	35	679032,86	8160499,56	-	-	No cq	ND
	36	678678,35	8160676,33	-	-	No cq	ND
	37	678855,85	8160388,48	-	-	No cq	ND
	38	679284,33	8160204,49	-	-	No cq	ND
	39	679737,29	8161727,32	-	-	No cq	ND
**North**	40	-16620154	-49.294.576	-	-	No cq	ND
	41	16.612.873	-49.299.803	-	-	No cq	ND
	42	-16.644.245	-49.229.127	+	-	34.21	105
	43	-16.643.354	-49.231.684	-	-	No cq	ND
	44	-16.618.823	-49.217.875	+	-	28.78	2442

^a^Geographic coordinate; ^b^Geographic coordinate; ^c^The viral load obtained and its number of copies determined by the standard Gblock curve; **CHIKV:** chikungunya virus; **ZIKV:** Zika virus; **Cq:** amplification cycle value obtained by RT-qPCR.

The transmission of arboviruses by the urban vector *A. aegypti* represents an important public health challenge because of its ability to cause diseases with significant social and economic impacts. The transmission of arboviruses occurs horizontally in competent mosquitoes, where the virus must be able to infect different anatomical regions of the vector, including overcoming the physical barriers of the midgut, and multiplying in secondary organs, such as the ovaries and salivary glands, present in the head and thorax of the mosquito. When a blood meal is obtained, the mosquito transmits the virus to another vertebrate^26^. The detection of CHIKV and ZIKV in the head and thorax pools suggests viral replication in the salivary glands of mosquitoes; that is, the progeny resulting from vertical transmission are potentially infectious and capable of transmission after birth.

Another form of arbovirus transmission is vertical, in which eggs are infected during oviposition, or transovarial transmission by the infected female mosquito^27^. This mechanism plays an important role in the persistence, dissemination, and maintenance of the virus in nature between inter-epidemic periods, as eggs can persist in the environment under adverse conditions, and in favorable environmental conditions, they hatch and generate infected progeny^28^.

Vertical transmission in *A. aegypti* has already been identified for the arboviruses CHIKV^29^ ^,^ ^30^, DENV^13^ ^,^ ^31^ ^,^ ^32^, ZIKV^14^ ^,^ ^33^ ^,^ ^34^, and MAYV^13^ ^,^ ^22^ through the analysis of eggs, larvae, and males of *A. aegypti*, experimentally, using adult *A. aegypti* fed with infected blood and stimulated to spawn. Contaminated environments used as sources of oviposition for *A. aegypti* and *A. albopictus,* such as sewage and urine, can also contaminate the larvae and pupae of these insects, as reported for ZIKV^27^.

The number of DENV and CHIKV recorded cases in Goiânia, the capital of the state of Goiás, significantly increased in 2022 compared with that in previous years, especially 2020 and 2021, during which studies supported the hypothesis of underreporting of arboviruses starting in 2020, likely due to the impact of the pandemic caused by the Sars-CoV-2 virus^35^ ^,^ ^36^. In Table 3 we present the epidemiological data of the arboviruses DENV, ZIKV, and CHIKV from the last 5 years in the city of Goiânia, reported by the State Secretariat of Goiás^37^.

**TABLE 3:** Epidemiological data on arboviruses over the last 5 years.

	Reported Cases		
Year	DENV	CHIKV	ZIKV
2018	33327	67	377
2019	35512	65	123
2020	16241	16	0
2021	14280	141	1
2022	60413	1462	1
2023	19271	510	2

**DENV:** dengue virus; **CHIKV:** chikungunya virus; **ZIKV:** Zika virus.

**FIGURE 2:**
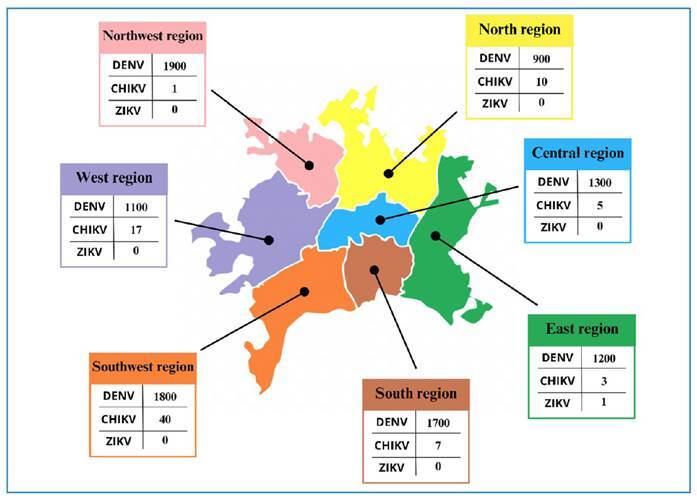
Distribution of all positive cases of DENV, CHIKV, and ZIKV in 2021 in Goiânia, Goiás.

In 2021, the municipality of Goiânia presented high risk and medium risk for the occurrence of DENV cases in all regions of the city, while CHIKV presented medium risk in the north regions, where a positive pool was detected, and in the southwest region. ZIKV cases decreased, with only one case in the city (Figure 2), which can be understood as an underreporting of cases or difficulty in diagnostic suspicion^21^.

Despite reports of vertical transmission of DENV in *A. aegypti,* both experimentally and in nature^32^ ^,^ ^38^, Goiânia has a high incidence of DENV cases (300 cases/100,000 inhabitants) in all regions of the city, especially in the northwest region^39^. In this study, no pools of *A. aegypti* infected with DENV were detected. This may be related to the fact that transovarial transmission occurs at a low frequency for DENV, not exceeding 20% in nature^40^ ^,^ ^41^, and that the highest rates of vertical transmission of DENV are associated with the *A. albopictus* rather than *A. aegypti* mosquito. Other studies also have not detected the presence of DENV in pools of *A. aegypti* in the Goiânia region^42^ ^.^

In the state of Goiás, as far as we know, there are no reports of the occurrence of OROV fever in humans; however, previous studies have identified the presence of antibodies to the OROV virus in primates captured in two parks in Goiânia^43^. The detection of antibodies against this virus in primates is important because of the possibility of its dissemination among these animals and potential for introduction among humans living in populated areas. Thus, in addition to the most frequent arboviruses in the city (DENV, CHIKV, and ZIKV), we performed tests to detect OROV. However, there were no positive pooled results for OROV because the main vectors related to this arbovirus are the mosquitoes *Culicoides paraensis* and *Culex quinquefasciatus* ^44^ ^,^ ^45^. There have been no reports of *A*. *aegypti* acting in the transmission of OROV, although it is a member of the genus. *Aedes serratus* has been identified as naturally infected by the virus^46^. During the egg collection period, the studied regions did not present a high OROV risk.

Cases of ZIKV and CHIKV infections are frequently underreported, mainly because infected individuals have mild symptoms or are asymptomatic. In addition, a lack of awareness, proper diagnosis, and efficient surveillance systems contribute to the underreporting of these viruses. This can lead to the mistaken perception that the epidemiological situation is under control when there is a continuous circulation of viruses in the mosquito population. Our findings underscore the importance of the ongoing surveillance and comprehensive monitoring of viral transmission. This highlights the need to strengthen public health systems, improve diagnostic methods, and promote collaboration among researchers, health professionals, and government officials to effectively detect and respond to mosquito-borne viral outbreaks.

The end of transmission and effective reduction of infestation by disease-transmitting mosquitoes in urban areas, including *A. aegypti*, is still a goal that is far from being achieved. Traditional methods have proven ineffective, and mosquito larvae show significant resistance to larvicides. Identification of sectors containing mosquitoes infected with arboviruses could be a valuable tool for disease control. This will enable public health authorities to make intensive efforts to reduce local vector populations, thereby reducing the potential for transmission by eliminating infected mosquitoes and shortening the infection cycle. This detection also opens the possibility of predicting transmission levels by estimating the percentage of infected mosquitoes within a given epidemiological week^47^.

## Conclusion

The vertical transmission of ZIKV and CHIKV arboviruses in *A. aegypti* mosquitoes, detected through the analysis of pools containing the head and thorax of *A. aegypti* females, indicates the formation of infectious offspring. Monitoring mosquitoes for the presence of arboviruses is crucial for preventing the spread of these infectious diseases because they typically undergo horizontal transmission, where insects transmit the viruses through blood feeding. Vertical transmission is an adaptive mechanism by which insects pass viruses to their offspring, facilitating their dispersal to urban areas. Mosquito surveillance programs can identify areas where infected mosquitoes are present, allowing public health authorities to implement targeted control measures, such as insecticide spraying, removal of stagnant water, and public education campaigns. Early detection and swift response can help prevent outbreaks and reduce arbovirus transmission, making mosquito monitoring essential for public health protection.
