# Supplementary material for: Detection of arboviruses in Aedes aegypti through transovarian analysis: A study in Goiânia, Goiás
Source: Rev Soc Bras Med Trop. 2024 Feb 23;57:e00400-2023. doi: 10.1590/0037-8682-0280-2023 (PMC10890825; doi:10.1590/0037-8682-0280-2023)
Supplement: Supplementary file 1 [file 1678-9849-rsbmt-57-e00400-2023-supp1.pdf]

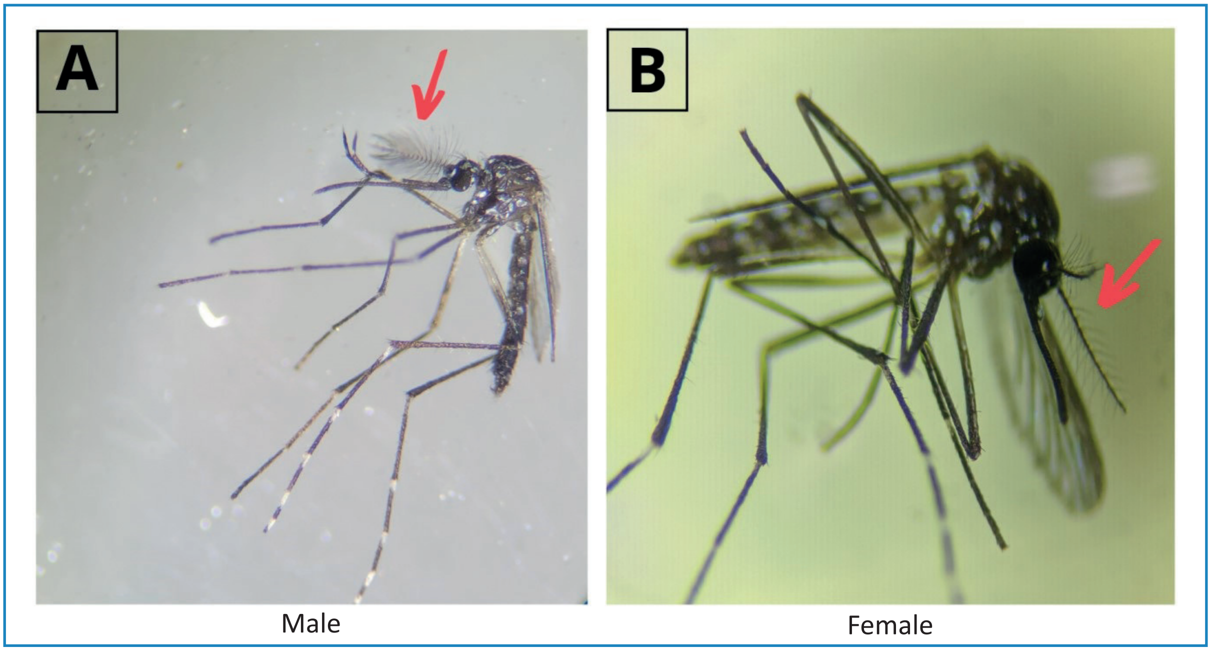

SUPPLEMENTARY FIGURE 1: Adult *A. aegypti* obtained by hatching eggs collected in the north, northwest, and southwest regions of Goiânia, Goiás. The females were separated and used to assemble the pools. Sexing was performed by analyzing the antenna of the insects, indicated by the arrow. (A) Male *A. aegypti*. (B) Female *A. aegypti*.
