# Supplementary material for: Detection of arboviruses in Aedes aegypti through transovarian analysis: A study in Goiânia, Goiás
Source: Rev Soc Bras Med Trop. 2024 Feb 23;57:e00400-2023. doi: 10.1590/0037-8682-0280-2023 (PMC10890825; doi:10.1590/0037-8682-0280-2023)
Supplement: Supplementary file 2 [file 1678-9849-rsbmt-57-e00400-2023-supp2.pdf]

SUPPLEMENTARY TABLE 1: Gblock sequences used for RT-qPCR.

**Gblock DENV, ZIKV, CHIKV, MAYV**	5’CCGCTGCCCAACACAAGGTGAAGCCTACCTTGACAAGCAATCAGACACTCAATATGTCTGCAAAAG<br>AACGTTAGTGGTTTTGGTTAGAGGAGACCCCTCCCTTACAAATCGCAGCAACAATGGGGGCCCAAGG<br>TGAGATGAAGCTGTAGTCTCACTGGAAGGACTAGAGGTTAGAGGAGACCCCCCCGAAATAAAAAAC<br>AGCATATTGACGCTGGGAAAGACCAGAGATCCTGCTGTCTCTTTTAAAGGGCAAACTCAGCTTCACAT<br>GCCGCTGTGATACAGTGGTTTCTTTTGTGCGAGGGCTACGTCGTTAAGAGAATAACGATGAGCCCAG<br>GCTTTTATAGACGACCTGCAGTCAGTGATGGCGACCCCGGACAATGAGTCACGGACATTTTGCCTTC<br>ACACAGATCAGACATGCAGGACTCCAGCTGAGGTGGCAGTCTATCA3’
**Gblock OROV and ACTIN**	5’GACCGACTACCTGATGAAGATCCTGACCGAGCGTGGCTACTCCTTCACCACTACCGCTGAACGTGA<br>AATCGTTCGTGACATTAAGGAGAAGCTGTGCTACGTCGCTCTGGACTTCGAGCAGGAAATGGCCACC<br>GCTGCTGCTTCCACCTCCCTGGAGAAGTCTTATGAACTTTTGACAAGTGCTCAATGCTGGTGTTGTTAG<br>AGTCTTCTTCCTCAACCAAAAGAAGGCCAAAGATGTCTTACGTAAGACATCGAGGCCCATGGTTGAC<br>CTTACTTTTGGTGGGGTCCAATTTGCAATGGTTAATAACCATTTCCCACAGTTCCAGTCGAATCCAGTG<br>CCGGACAACG3’

RT-qPCR: reverse-transcription quantitative polymerase chain reaction, **DENV:** dengue virus; **ZIKV:** Zika virus; **CHIKV:** chikungunya virus; **MAYV:** Mayaro virus; **OROV:** oropouche virus.
